# Mean Protein Evolutionary Distance: A Method for Comparative Protein Evolution and Its Application

**DOI:** 10.1371/journal.pone.0061276

**Published:** 2013-04-15

**Authors:** Michael J. Wise

**Affiliations:** School of Chemistry and Biochemistry, University of Western Australia, Crawley, Western Australia, Australia; University of Georgia, United States of America

## Abstract

Proteins are under tight evolutionary constraints, so if a protein changes it can only do so in ways that do not compromise its function. In addition, the proteins in an organism evolve at different rates. Leveraging the history of patristic distance methods, a new method for analysing comparative protein evolution, called Mean Protein Evolutionary Distance (MeaPED), measures differential resistance to evolutionary pressure across viral proteomes and is thereby able to point to the proteins’ roles. Different species’ proteomes can also be compared because the results, consistent across virus subtypes, concisely reflect the very different lifestyles of the viruses. The MeaPED method is here applied to influenza A virus, hepatitis C virus, human immunodeficiency virus (HIV), dengue virus, rotavirus A, polyomavirus BK and measles, which span the positive and negative single-stranded, doubled-stranded and reverse transcribing RNA viruses, and double-stranded DNA viruses. From this analysis, host interaction proteins including hemagglutinin (influenza), and viroporins agnoprotein (polyomavirus), p7 (hepatitis C) and VPU (HIV) emerge as evolutionary hot-spots. By contrast, RNA-directed RNA polymerase proteins including L (measles), PB1/PB2 (influenza) and VP1 (rotavirus), and internal serine proteases such as NS3 (dengue and hepatitis C virus) emerge as evolutionary cold-spots. The hot spot influenza hemagglutinin protein is contrasted with the related cold spot H protein from measles. It is proposed that evolutionary cold-spot proteins can become significant targets for second-line anti-viral therapeutics, in cases where front-line vaccines are not available or have become ineffective due to mutations in the hot-spot, generally more antigenically exposed proteins. The MeaPED package is available from www.pam1.bcs.uwa.edu.au/~michaelw/ftp/src/meaped.tar.gz.

## Introduction

**Figure 1:**
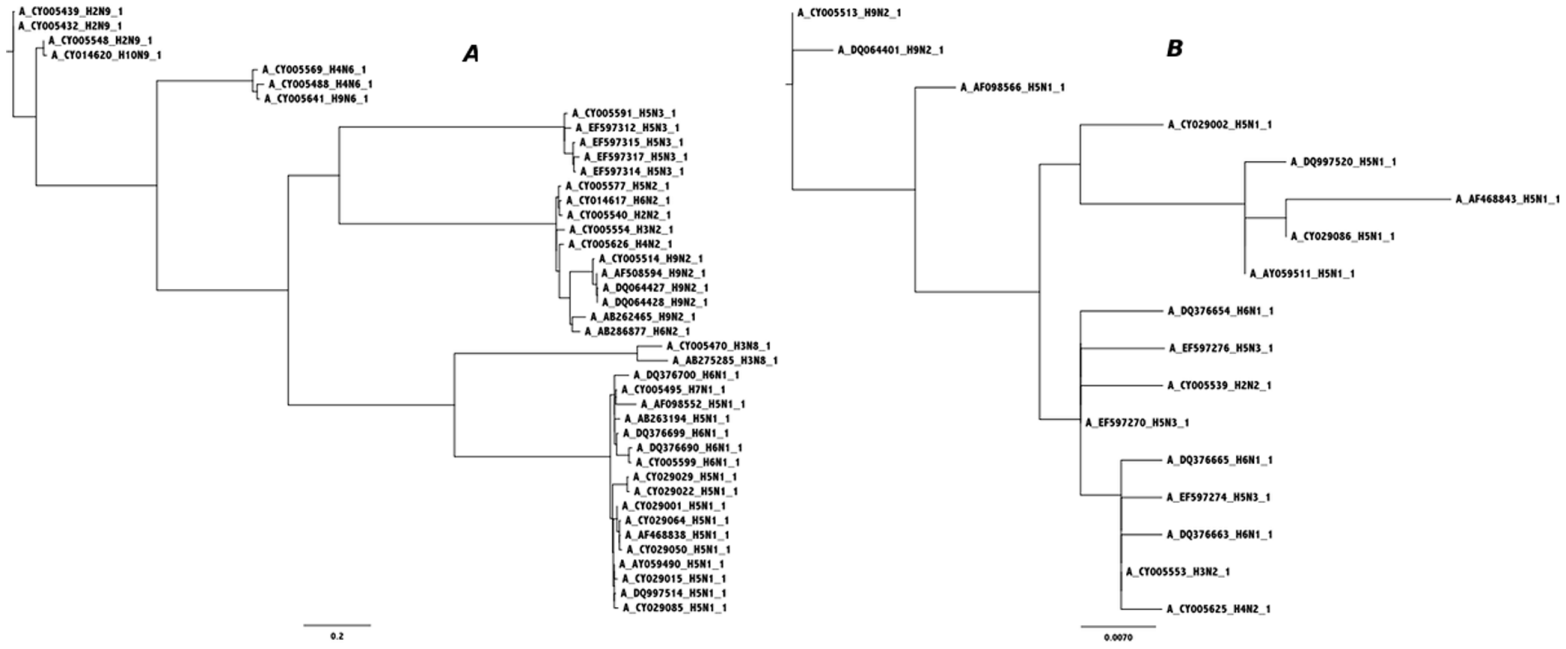
Phylogenetic trees based on avian influenza M1 matrix and neuraminidase proteins. Phylogenetic trees have been created based on small sets of M1 matrix proteins (a) and corresponding neuraminidase proteins (b) taken from complete influenza proteomes. The trees were created using Phyml and the figures drawn using FigTree. Notice that the neuraminidase tree forms clades largely corresponding to influenza type. Notice also that the scale bar is much larger for the neuraminidase tree.

Proteins are under tight evolutionary constraints because they are the main agents of the processes required by all organisms. Therefore, if a protein changes it can only do so in ways that do not compromise its function. This is particularly true for the small viruses that lack the levels of redundancy available to their eukaryote (or even bacterial) hosts. Viruses make up for this by having high levels of adaptability, driven by high mutation rates. For example, while *E. coli* has as mutation rate of 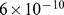 muations/bp/replication [1], the dsDNA virus Bacteriophage T2’s rate is 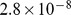 [1] and RNA viruses have mutation rates in the range 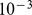 to 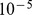 [2]. However, not all the genes in a virus – or any other organism – vary at the same rate. In phylogenetic analyses one, or perhaps several, known genes or proteins from a range of species are typically used to make inferences about the evolutionary histories of the source species, or to calculate the genetic distances between species. Indeed, these efforts predate the use of DNA or protein sequence data, e.g. Faith (1992) [3]. Differential rates of evolution bedevils the choice of which genes to use for these analyses; see, for example, the discussion in D’Erchia et al. (1996) [4]. However, I propose to turn this sort of analysis on its head and instead use comparisons of the rates of evolution evident across the proteins from a single species to yield significant insights about those proteins. To motivate the description of the method, Figure 1 shows phylogenetic trees that have been created based on an initial set of 43 avian influenza M1 matrix proteins and a set of 43 neuraminidase proteins from the same isolates. From the initial sets, 100% identical proteins were deleted and multiple sequence alignments were created from the remaining 17 (M1) and 43 (neuraminidase) sequences using Muscle [5]. Phyml version 3.0 [6], bootstrapped 100 times, was then used to create phylogenetic trees. The first thing to note from Figure 1b (neuraminidase) is that the clades formed during tree construction correspond to the designated neuraminidase types. The more important thing to note in each of the graphs is the scale bar; For the M1 set the scale bar is 0.007, while for the neuraminidase set the scale bar is 0.2. In other words, if the neuraminidase tree were drawn to the same scale as the M1 tree it would be 29 times larger. The inference is that despite strong purifying selection operating on both proteins, neuraminidase functional requirements necessitate a much higher evolutionary rate and hence greater protein diversity (43 unique neuraminidase proteins versus 17 for M1 protein, in this small example). In other words, we see in neuraminidase a greater resistance to evolutionary pressure. If evolutionary pressure is seen as the centripetal force that restricts genomic change through purifying selection – here measured at the protein level – then resistance to evolutionary change is the countervailing (centrifugal) force for change.

Leveraging the large numbers of isolates from single viral species that are increasingly becoming available, using a phylogenetic-tree based method described below we can now examine the differential evolutionary rates of proteins within a single species. The underlying idea is not new; minimising patristic distance – the distance between taxa in phylogenetic trees – underlies the Neighbour-Joining algorithm [7], and Farris (1972) [8] proposes the use of patristic distances (which he calls patristic differences) from taxa to a putative root taxon as a measure of evolution in a single protein. Here the idea will be extended to facilitate the comparison of proteins from isolates of a single species, and ultimately comparisons between species. The new method, called Mean Protein Evolutionary Distance (MeaPED), fits into the considerable literature on protein evolution; see, for example the review Pàl et al. (2006) [9]. However, the preeminent current method for measuring evolutionary pressure is the ratio of nonsynonymous to synonymous substitutions (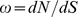)– also known as Ka/Ks – which is discussed in Yang (2006) [10]. The MeaPED method will be compared with 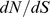. Despite misgivings expressed in Kryazhimskiy and Plotkin (2008) [11] about the applicability of 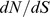 to single populations, at least for the viral species discussed below the results suggest that comparisons with this method are reasonable.

### The New Approach

Mean Protein Evolutionary Distance (MeaPED) is a way of measuring differential resistance to evolutionary pressure across sets of proteins from a single viral species. The MeaPED process begins with collecting complete proteomes from isolates of the species of interest, such as influenza A virus, where the aim is to sample as much of the sequence diversity within the species as possible. The protein sequences are grouped, e.g. haemagglutinin (HA), neuraminidase (NA), etc., An important technical point is that care must be taken to ensure that each set contains the same gene from the different isolates, i.e. true orthologues, and that paralogues are only found in their own sets, e.g. only E1 in the E1 set, only E2 in the E2 set (from hepatitis C virus). (Paralogues arise due to gene duplication; alpha and beta haemoglobin are a well known example of paralogous genes in vertebrates. Gene duplications are common in the large dsDNA viruses, e.g. mimivirus [12].) In practice, this means the one-to-one mapping of positional (i.e. syntenic) orthologues [13].

For each protein, an unrooted phylogenetic tree is constructed from the set of unique sequences. The choice of phylogenetic tree building engine and amino acid substitution matrix will be discussed below. For each leaf node (i.e. taxon) in the resulting phylogenetic tree the mean patristic distance is computed between it and every other leaf node. Then the mean of these means is calculated – a single value representing the mean evolutionary distance for that protein computed across the set of unique sequences. An adjusted mean-of-means (AMM) is also computed, where the denominator is the original count of sequences rather than the final count of sequences once duplicates have been deleted. This reflects the fact that duplicate sequences add no new information, i.e. no additional variability. While a reduced number of sequences due to deletion of duplicates can imply over-sampling of that strain and gene during sample collection, it can also imply that there is a limited number of amino acid encodings of the corresponding protein which are viable, i.e. still are able to perform that protein’s function. As a final step, the adjusted mean of means for a set of sequences is divided by the median input sequence length, times 100, giving the adjusted mean distance per 100 aa (AMM100), to facilitate comparisons between proteins with different average sequence lengths. Sorting the list of proteins by decreasing AMM100 value reveals both evolutionary hot-spot proteins (higher average evolutionary rates) and evolutionary cold-spot proteins.

One issue with the use of patristic distances to measure protein evolution, e.g. as proposed in Farris (1972) [8], is that branches close to the designated root are counted multiple times. By averaging patristic distances using each taxon in turn as the root, there is no difference in the number of times leaf branches are counted. However, internal branches will be counted more times than leaf branches, particularly branches linking “clades” (i.e. subtrees of highly similar proteins that are substantially different to proteins in other subtrees). The effect – a weighted mean, with the impact blunted by averaging – is desirable because it is the internal branch lengths, particularly those between “clades”, that reflect a requirement in certain proteins for higher evolutionary rates despite strong purifying selection. By contrast, using a simple mean of patristic distances, for example, would result in evidence from the small number of significant internal branches (inter-clade) being lost among the much larger number of intra-clade distances.

## Results

Table 1 below shows the results of a MeaPED analysis of human influenza A virus, hepatitis C virus (type 1), human immunodeficiency virus type 1 (subtype b), dengue virus (type 1), measles, polyomavirus BK and rotavirus A. (The analyses of these plus swine and avian influenza A virus, HIV1 subtypes c and d, dengue virus types 2,3,4 and hepatitis C virus types 2,3,4,6 comprise Table S1.) The viral species, spanning the positive and negative single-stranded, doubled-stranded and reverse transcribing RNA viruses, and double-stranded DNA viruses, are summarised in Table 2.

**Table 1:** Protein Evolutionary Distances - Initial and Final counts of sequences following deletion of duplicates, Mean PED, Adjusted Mean PED and Adjusted Mean PED per 100 aa, and mean 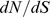.

			Dengue virus type 1				
Protein	Med.Len.	Init N	Final N	Mean	Adj. Mean	Adj. Mean per 100 aa	dnds
NS2a	654.0	651	323	0.0394	0.0196	0.0090	0.0925
M	221.3	651	186	0.0219	0.0063	0.0086	0.0622
NS4a	381.0	649	246	0.0254	0.0096	0.0076	0.0592
C	300.0	651	149	0.0297	0.0068	0.0068	0.1883
NS2b	390.0	651	258	0.0219	0.0087	0.0067	0.0415
2K	69.0	622	60	0.0158	0.0015	0.0066	0.0336
NS1	1056.0	651	392	0.0288	0.0174	0.0049	0.0560
NS4b	747.0	651	304	0.0158	0.0074	0.0030	0.0383
E	1489.4	651	435	0.0195	0.0130	0.0026	0.0525
NS5	2697.0	633	472	0.0212	0.0158	0.0018	0.0512
NS3	1857.0	651	465	0.0131	0.0093	0.0015	0.0281
			**Hepatitis C virus type 1**				
**Protein**	**Med.Len.**	**Init N**	**Final N**	**Mean**	**Adj. Mean**	**Adj. Mean per 100 aa**	**dnds**
p7	189.0	804	749	0.3524	0.3283	0.5211	0.1158
NS2	651.0	804	778	0.5698	0.5514	0.2541	0.0974
E1	576.1	804	764	0.4907	0.4663	0.2428	0.0878
NS4a	162.0	804	733	0.1388	0.1265	0.2343	0.0698
E2	1089.3	804	780	0.7518	0.7294	0.2009	0.0761
F	485.0	630	581	0.3161	0.2915	0.1810	4.6603
NS4b	783.0	804	774	0.3403	0.3276	0.1255	0.0397
NS5a	1342.8	804	779	0.4860	0.4709	0.1051	0.0859
NS5b	1769.7	743	733	0.2824	0.2787	0.0471	0.0866
NS3	1893.0	804	782	0.3045	0.2962	0.0469	0.0349
C	573.0	804	754	0.0481	0.0451	0.0236	0.0482
			**HIV1 subtype b**				
**Protein**	**Med.Len.**	**Init N**	**Final N**	**Mean**	**Adj. Mean**	**Adj. Mean per 100 aa**	**dnds**
VPU	247.9	1018	700	0.5390	0.3706	0.4575	0.2750
VPR	290.9	1001	692	0.5365	0.3709	0.3863	0.3525
NEF	626.6	795	624	0.9122	0.7160	0.3459	0.3616
TAT	306.1	1021	722	0.4233	0.2993	0.2963	0.9970
REV	351.0	1023	724	0.3848	0.2723	0.2348	0.8367
VIF	579.2	1016	754	0.3996	0.2966	0.1545	0.4192
ENV	2575.5	975	875	0.7628	0.6845	0.0799	0.4389
GAG	1509.0	996	810	0.3543	0.2881	0.0575	0.1367
POL	3015.8	984	896	0.2816	0.2564	0.0256	0.1336
			**Human Influenza**				
**Protein**	**Med.Len.**	**Init N**	**Final N**	**Mean**	**Adj. Mean**	**Adj. Mean per 100 aa**	**dnds**
HA	1697.3	3368	2159	1.5015	0.9625	0.1701	0.1042
NA	1407.3	3357	1777	1.1722	0.6205	0.1323	0.1258
PB1_F2	237.8	1932	344	0.6240	0.1111	0.1235	1.3072
NS1	680.7	3364	1140	0.2652	0.0899	0.0391	0.2740
M2	290.8	3359	539	0.1301	0.0209	0.0215	0.4055
NS2	363.0	3349	624	0.0995	0.0185	0.0153	0.1124
NP	1494.0	3345	1656	0.1308	0.0648	0.0130	0.0827
PA	2148.0	3339	2090	0.0691	0.0432	0.0060	0.0640
M1	756.0	3363	913	0.0542	0.0147	0.0058	0.0756
PB2	2277.0	3338	2190	0.0624	0.0409	0.0054	0.0590
PB1	2271.1	3343	2143	0.0448	0.0287	0.0038	0.0496
			**Measles**				
**Protein**	**Med.Len.**	**Init N**	**Final N**	**Mean**	**Adj. Mean**	**Adj. Mean per 100 aa**	**dnds**
V	900.4	23	14	0.0407	0.0248	0.0083	1.0998
C	561.0	30	16	0.0276	0.0147	0.0079	0.2756
P	1524.0	34	23	0.0387	0.0262	0.0052	0.6530
N	1578.0	34	22	0.0236	0.0153	0.0029	0.2288
M	1008.0	33	22	0.0141	0.0094	0.0028	0.3392
H	1854.5	34	23	0.0246	0.0166	0.0027	0.7055
F	1657.8	34	22	0.0126	0.0081	0.0015	0.4244
L	6552.0	34	29	0.0119	0.0101	0.0005	0.1804
			**Polyomavirus**				
**Protein**	**Med.Len.**	**Init N**	**Final N**	**Mean**	**Adj. Mean**	**Adj. Mean per 100 aa**	**dnds**
AGNO	199.3	528	38	0.0394	0.0028	0.0043	0.4549
VP1	1089.0	530	124	0.0425	0.0099	0.0027	0.1217
VP3	699.0	528	52	0.0334	0.0033	0.0014	0.1531
VP2	1056.0	528	57	0.0238	0.0026	0.0007	0.1554
ST	519.0	530	48	0.0109	0.0010	0.0006	0.1269
LT	2087.6	526	138	0.0089	0.0023	0.0003	0.0328
			**Rotavirus**				
**Protein**	**Med.Len.**	**Init N**	**Final N**	**Mean**	**Adj. Mean**	**Adj. Mean per 100 aa**	**dnds**
VP7	977.2	135	98	0.2149	0.1560	0.0478	0.0609
NSP1	1458.9	129	94	0.2253	0.1641	0.0338	0.1122
NSP4	525.0	134	84	0.0534	0.0335	0.0191	0.1037
NSP5	591.3	131	67	0.0534	0.0273	0.0139	0.1339
NSP3	931.7	118	78	0.0466	0.0308	0.0099	0.0613
NSP2	951.0	122	71	0.0488	0.0284	0.0090	0.0461
VP4	2325.2	135	102	0.0907	0.0685	0.0088	0.0401
VP3	2505.0	130	98	0.0624	0.0471	0.0056	0.0537
VP6	1189.7	130	82	0.0172	0.0109	0.0027	0.0131
VP2	2675.1	131	98	0.0307	0.0230	0.0026	0.0214
VP1	3260.5	135	113	0.0246	0.0206	0.0019	0.0345

**Table 2:** List of Viral Species Examined in this Study.

Species	Type	Genome format	N proteins
Influenza virus	Negative Sense ssRNA	Segmented linear	11
Measles	Negative Sense ssRNA	Single linear	8
Hepatitis C virus	Positive Sense ssRNA	Single, linear forming polyprotein	11
Dengue virus	Positive Sense ssRNA	Single, linear forming polyprotein	11
HIV 1	Positive Sense RNA Reverse Transcribing	Single linear	9
Rota virus	dsRNA	Segmented linear	11
Polyoma virus	dsDNA	Single Circular	6

### Influenza

In Table 1, the proteins for the different viruses are sorted by decreasing AMM100 value (column 7), with 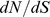 values for the corresponding genes being found in column 8. Looking in Table 1 specifically at influenza virus, the fact that the antigenically exposed haemagglutinin (HA) and neuraminidase (NA) show the most variation comes as no surprise. The appearance of PB1-F2 as an evolutionary hot-spot in human (and also avian and swine) influenza is more controversial. First described in Chen et al. (2001) [14], PB1-F2 is understood to induce apoptosis through interactions with mitochondrial membrane adenine nucleotide translocator 3 (ANT3) and outer mitochondrial membrane voltage-dependent anion channel 1 (VDAC1) [15]. However, there are no single nucleotide polymorphisms (SNPs) in the exons of VDAC1 and at most one in the exons of ANT3 (see Methods), so variability in these interacting proteins cannot be driving the evolution of PB1-F2. PB1-F2 has also been found to interact with PB1, a subunit of the RNA polymerase complex [16]. Here again, Table 1 shows that there is two orders of magnitude less variability in PB1 than in PB1-F2. PB1-F2 has recently been shown to be natively unfolded, form amyloids and be able to perforate cell membranes [17]. On the other hand, it is argued in Trifonov et al. (2009) [18] that because PB1-F2 accumulates stop-codons and is therefore often truncated – and generally shows little evidence of selection – it does not play a significant evolutionary role. Clearly further research is needed to determine the role of PB1-F2 *in vivo*. Looking further down the AMM100 list for influenza virus, the NS1 protein AMM100 score is an order of magnitude less than haemagglutinin, neuraminidase and PB1-F2, and is known to play a role in immune evasion [19]. At the bottom of the list are polymerase proteins PB1 and PB2, whose AMM100 scores are an order of magnitude less again. It is also of interest to note that AMM100 values are higher in avian and swine influenza than in human influenza, indicating greater resistance to evolutionary pressure.

### Hepatitis C Virus

Scanning the AMM100 values for hepatitis C virus it is clear that the resistance to evolutionary pressure on its genes is generally higher than for influenza virus, with the top three values being for p7, NS2 and envelope protein E1. The p7 is a short (63 aa) non-structural protein that oligomerises and becomes resident in the endoplasmic reticulum. It has been found to have ion channel activity and to be involved in the release of infectious hepatitis C virus [20]. (Indeed, VPU – another viroporin found in HIV – has the highest AM100 score for all three of the HIV1 subtypes examined in this study.) Envelope glycoproteins E1 and E2 dimerise to gain entry into host cells [21]. The E1, E2 and p7 proteins have also been shown to be immunogenic [22], [21]. In view of that it is interesting to note the lower AM100 value for the longer E2 protein (363 aa) versus the shorter E1 protein (192 aa), though their adjusted mean scores are similar, suggesting that while some portions of the E2 protein provide evidence of higher evolutionary rate, other parts are relatively conserved. The evidence for NS2 as an evolutionary hot-spot for hepatitis C virus is mixed, for although it has a high AMM100 score for hepatitis C virus type 1 and type 4, it has a more middling score for the other hepatitis C virus types. While it has been known for some time that NS2 works cooperatively with NS3 to cleave the peptide bond linking NS2 and NS3, the function of the mature NS2 protein is still uncertain. However, NS2 has been shown *in vitro* to inhibit host cell gene expression [23] and to interfere with CIDE-B induced, caspase mediated apoptosis [24]. At the other end of the scale, NS5b (RNA-dependent RNA polymerase), NS3 (serine protease/helicase) and the C (core) protein – involved in capsid formation – [25] are cold spot proteins across the hepatitis C virus types.

### Fast and Slow: HIV versus Dengue Virus and Polyomavirus

Analysis of HIV1 reveals a very similar picture to hepatitis C virus – even the most slowly varying protein, the POL reverse transcriptase/integrase polyprotein, is evolving at a rate similar to the mid-range NS1 of influenza, while most of the remaining genes are evolving at a rate similar to, or faster than, influenza’s haemagglutinin or neuraminidase. By contrast, dengue virus and polyomavirus present the opposite picture. Being a mosquito-borne pathogen, dengue virus has to survive in both the mosquito and human hosts, implying a double constraint on the evolution of its proteins, which is reflected in AMM100 values that are comparable with M1 at the highest and then drop an order of magnitude. The polyomavirus proteins have a similar AMM100 score profile to dengue virus, though for a somewhat different reason. After primary acute infection, the dsDNA polyomavirus can persist for a along period in its host, only reappearing in immunocompromised hosts [26]. Indeed, long term persistence of dsDNA viruses in their hosts, to which they are generally closely adapted, is a hallmark of these viruses and it has been argued that this persistence has contributed to virus-host coevolution [27].

### Analysis of the MeaPED Method

An obvious question is whether MeaPED analyses are robust or dependent on the choice of phylogenetic tree building application, and then the choice of amino acid substitution matrix. One practical constraint is that whichever phylogenetic-tree building method is chosen, it must be able to deal with large data-sets; the largest used here (human influenza) has more than 3,300 sequences for each protein. One such application is the Maximum Likelihood method Phyml version 3.0 [6], which was used for the analyses described above, in combination with the default LG amino acid substitution matrix [28]. To test the robustness of MeaPED, the Neighbour-Joining applications Protdist (default JTT amino acid substitution matrix) and Neighbor (from the Phylip suite [29]) were substituted for Phyml and the experiments were repeated. Spearman Rank Correlations where computed comparing the Phyml-based and Neighbor-based calculations for each species/subtype across the sets of proteins. The coefficients of determination (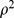) and the associated p-values are shown in Table 3. The results indicate that the MeaPED method is largely independent of the choice of phylogenetic-tree building method (and amino acid substitution matrix).

**Table 3:** Spearman Rank Correlation of MeaPED Analyses Undertaken via Phyml and Neighbor.

Species	N proteins	Spearman *ρ* ^2^	p-value
DENV1	11	0.964	8.40e−08
DENV2	11	0.964	8.40e−08
DENV3	11	0.982	3.706e−09
DENV4	11	0.982	3.76e−09
HIV1b	9	0.751	2.50e−03
HIV1c	9	0.871	2.36e−04
HIV1d	9	0.934	2.16e−05
HCV1	11	0.894	1.12e−05
HCV2	11	0.946	5.14e−07
HCV3	11	0.946	5.14e−07
HCV4	11	1.000	1.29e−13
HCV6	11	0.982	3.76e−09
Avian Influenza	11	0.995	7.46e−12
Swine Influenza	11	1.000	1.29e−13
Human Influenza	11	0.964	8.40e−08
Measles	8	0.862	0.001
Polyomavirus	6	1.000	1.46e−05
Rotavirus A	11	1.000	1.29e−13

A second question is how Mean Protein Evolutionary Distance compares with the standard approach: the ratio of nonsynonymous to synonymous substitutions, 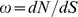. To test this, the rankings of proteins based on AMM100 values were compared using Spearman Rank Correlations across the different virus subtypes. A similar analysis was undertaken with the orderings based on the mean 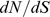 values computed across the corresponding genes. Such comparisons are valid, for although the evolutionary rates of orthologous genes can in general vary between species, in this case the analysis is based on the identical genes coming from different isolates of the same species, so therefore subject to the same functional constraints. This effect is strengthened because positional orthologues are subject to tighter evolutionary constraints [13]. The approach appears to be borne out by the results, shown in Table 4, where the ranking of proteins by AMM100 score across, for example, HIV subtypes has 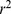 of 0.83 while the ranking of the corresponding genes by 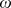 value has 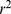 of 0.86. On the other hand, Penn et al (2008) [30] would seem to question this assumption, with the paper providing evidence that different HIV1 subclades can have different evolutionary rates – what they call “rate shifts”. However, looking at the extended results in Table S1 you can see that protein-for-protein, HIV1 subtype c, for example, has a higher AMM100 value than the corresponding rank in HIV1 subtype d. In other words, for this hyper-mutator virus, the evolutionary rate for a protein does change between subtypes, but so too do the other proteins in the corresponding subtypes, preserving the ordering. This is implicit in Table 1 from [30], where the ranking of Proportion of Shifting Sites approximately follows that seen due to AMM100 values, with Pol having the lowest proportion (i.e. being the most constrained) and Vpu having the highest proportion. In this light, a statistical test based on ranking, rather than absolute values, is appropriate. Turning to the AMM100 versus dN/dS based rankings, shown in Table 4, for the dengue virus subtypes, the MeaPED approach was somewhat better, while for the different influenza host species subtypes, the 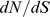 approach was somewhat better. For the HIV subtypes the orderings of the genes due to MeaPED and 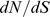 were equally consistent. However, for the Hepatitis C subtypes the MeaPED approach produced significantly more consistent results.

**Table 4:** Comparing the consistency of AMM100 and 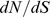 values across virus subtypes.

	Dengue virus	(*N* = 4 *types*)	
Method	mean *r* ^2^	Stouffer MST	Fisher MST
AMM100	0.790	4.14e−10	1.93e−09
dnds	0.615	7.47e−07	5.55e−07
	**Human immunodeficiency virus type 1**	**(** ***N*** ** = 3 ** ***subtypes)***	
**Method**	**mean ** ***r*** **^2^**	**Stouffer MST**	**Fisher MST**
AMM100	0.831	3.82e−07	9.85e−07
dnds	0.857	3.21e−07	6.23e−07
	**Hepatitis C virus**	**(** ***N*** ** = 5 ** ***types*** **)**	
**Method**	**mean ** ***r*** **^2^**	**Stouffer MST**	**Fisher MST**
AMM100	0.916	1.018e−20	1.43e−19
dnds	0.60	1.35e−07	1.07e−06
	**Influenza virus**	**(** ***N*** ** = 3 ** ***hostspecies*** **)**	
**Method**	**mean ** ***r*** **^2^**	**Stouffer MST**	**Fisher MST**
AMM100	0.900	5.59e−10	1.65e−09
dnds	0.946	1.57e−12	4.75e−12

A third question is whether there is a correlation between AMM100 (or 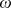) values and the number of sequences in the input set. This can be investigated by observing that in this study data is provided for the subtypes of certain viruses: influenza, hepatitis C virus, dengue virus and HIV, and the different subtypes are represented by different numbers of sequences. For each gene in a given virus, the AMM100 (or 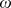) values can be correlated across virus subtypes with the final counts of sequences after the deletion of duplicate sequences, 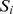. On that basis, AMM100 scores for 8 out of 11 dengue virus genes were positively correlated with 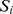 across 4 virus subtypes, while scores for 8 out of 9 HIV genes were positively correlated across 3 virus subtypes and scores for 7 out of 11 hepatitis C virus genes were positively correlated across 5 virus subtypes. On the other hand, only 3 out of 11 influenza AMM100 scores were positively correlated with 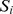 across 3 virus subtypes. Given that the number of virus subtypes is small – N = 3 (influenza and HIV), N = 4 (dengue virus) and N = 5 (hepatitis C virus) – and therefore the possibility that the lists of values can be correlated by chance, together with the fact that each species had some genes yielding opposite correlations, it can be assumed that there is no systematic correlation between AMM100 values and the counts of sequences being examined. (Analysis based instead on 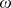 yields a similar conclusion.)

A final question is whether there is a relationship between MeaPED scores and the lengths of the input sequences, represented by the median input sequence length for each species subtype. It is plausible that an inverse relationship may exist – longer sequences attracting lower MeaPED scores – because, assuming globular structures, larger proteins will have more of their residues buried and buried residues are known to mutate more slowly than surface residues, particularly residues occurring in loops. To investigate this, for each virus subtype a linear regression was done between the set of Mean or AMM100 scores and the corresponding median input sequence lengths. Of the 18 virus subtypes, 12 returned a negative correlation between median input sequence length and mean MeaPED score. However, none of these was significant, the most significant being measles (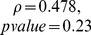) and polyomavirus (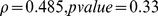). Linear regression models involving AMM100 produced more significant results – dengue virus types 1 and 2, HIV2 subtypes b and c and hepatitis C virus (all types) were significant at the 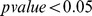 level, but that is to be expected because computation of AMM100 involves the median input sequence length. (Comparisons involving 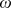 yielded similar results to those involving the mean MeaPED score.) In summary, there is a small affect due to input sequence length, but it is not significant.

## Discussion

### Likely Roles of Hot Spot and Cold Spot Proteins

One observation evident from the Results is that relative hot spot proteins are likely to interact with the host. Examples include: hemagglutinin (influenza) and viroporins agnoprotein (polyomavirus), p7 (hepatitis C) and VPU (HIV). For measles, the protein with the highest AMM100 score is the V protein, which is known to inhibit alpha interferon signalling through a number of interactions, including acting as a decoy substrate for I
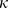
B kinase 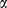, preventing phosphorylation of IFN regulatory factor 7 [31]. In this context it is also interesting to contrast human influenza hemagglutinin – AMM100 value of 0.1701, and at the top of the list of influenza virus AMM100 values – with measles virus hemagglutinin, which has an AMM100 value of 0.0027 and near the bottom of the measles virus AMM100 values. Unlike influenza virus, which has separate neuraminidase (NA) and hemagglutinin (HA) proteins, paramyxoviruses, including measles, have the two functions performed by the same, HN protein. However, in the measles virus, the protein lacks neuraminidase activity (and is therefore designated “H”). It also does not bind sialic acid and the binding pocket appears enlarged [32]. Instead, the H protein binds signalling lymphocyte activation molecule (SLAM, also called CD150) and CD46 [33]; CD46 regulates complement activation while SLAM triggers a cascade that results in cytokine release, including IL4 and IL13. In other words, rather binding sialic acid as does influenza hemagglutinin, and inducing a strong immune response [34], measles virus H protein binds to SLAM or CD46, thereby gaining entry to cells and also performing immunosuppression by acting as a inhibitor of those key proteins [33].

MeaPED analysis can also highlight cold spot proteins. These include the RNA directed RNA polymerase proteins PB1 and PB2 (influenza), NS5 (dengue virus), L (measles virus), NS5b (hepatitis C virus), and VP1 (rotavirus), and internal serine protease NS3 (dengue and hepatitis C). The POL protein from HIV combines an internal protease with a reverse transcriptase/integrase. The theme that emerges is that, in contrast to hot spot proteins, cold spot proteins are internal to the working of the virus. While the identification of hot-spot proteins can yield useful biological insights, the identification of cold-spot proteins, on the other hand, can tell us which proteins are less likely to evolve should they be targeted by anti-viral therapeutics. For this analysis, the adjusted mean of means (AMM) score is sometimes more useful than the AMM100 score. For example, near the bottom of the Table 1 list for influenza A virus is the matrix protein M1, whose AMM score is two orders of magnitude less than the top three in the list. Multiple sequence alignment of the M1 protein reveals quite long peptides (20–40 aa) which are identical (or very nearly so) across the large human, swine and avian data-sets, but which may not be “drugable” in the conventional sense. While any anti-viral therapy will act as a selective agent and induce increased viral evolutionary rates, if M1 can be targeted, e.g. through peptide ligands such as Phylomers [35], the implication is that M1 will evolve far less rapidly than haemagglutinin or neuraminidase, which are the targets of vaccines and drugs such as Relenaza (Zanamivir) or Tamiflu (Oseltamivir).

### Comparison with 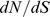


While the primary intention of using 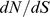 has been to rank evolutionary rates, a general observation from the 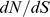 data presented here is that, with the exception of HIV, the vast majority of the genes have average 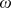 values of 0.1 or less, placing them in the range – according to Kryazhimskiy and Plotkin (2008) [11] – where strong purifying selection can be assumed, justifying the assumption that the evolution of proteins, at least for these viruses, is tightly constrained. The hint for why this might be the case comes from two concepts developed in that paper: evolution over long versus short time-scales and silent (or non-silent) mutations versus fixations. Given short viral doubling times and high mutation rates, one can assume that a wide range of possible encodings for a protein will have been tried over what is a relatively short chronological time, but representing many generations. However, functional constraints on the proteins will mean that only the small number of encodings leading to viable proteins will be fixed in a population. This is very evident, for example, in neuraminidase from influenza, where the different clades are very distinct. It is therefore plausible that the different clades represent quasispecies [2]. Both the average 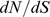 computations and the MeaPED analyses involve all-against-all comparisons, which emphasise the distances between the quasispecies, as most of the comparisons will be between sequences from different clades/quasispecies rather than sequences from the same clade/quasispecies.

### In Summary

These examples illustrate that the Mean Protein Evolutionary Distance approach is a robust method that concisely and consistently captures an important facet of viral protein function through their differing responses to evolutionary pressure. In doing so it is at least as effective, if not better than, the equivalent computation using average 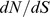 values, does not suffer from issues of interpretation highlighted by Kryazhimskiy and Plotkin (2008) [11] and, unlike the 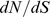 method, the MeaPED method is able to make use of data from gaps in the underlying multiple-sequence alignments, neglect of which can produce less accurate trees [36]. The MeaPED method is also considerably quicker, particularly for large data-sets. Finally, although MeaPED analysis has to date only been used on viral data-sets, with the increasing number of isolates from different microbial genomes being sequenced the data is becoming available for the method to also be applied to proteomes of species from other kingdoms.

## Methods

### Sources of Sequences Used

Seven species were examined for this study: human, swine and avian influenza A virus, hepatitis C virus (types 1,2,3, 4 and 6), human immunodeficiency virus type 1 (subtypes b, c and d) and dengue virus (types 1,2,3 and 4), measles, polyomavirus BK and rotavirus A. There were too few hepatitis C virus type 5 sequences for analysis to be carried out. In each case, complete coding-sequence (CDS) sets were obtained for each isolate of the given species and type.

The influenza data-sets came from the Influenza Virus Resource at NCBI [37] http://www.ncbi.nlm.nih.gov/genomes/FLU/. Coding sequences for different influenza types, different host species and different geographic locations can be obtained via the web site. Because a very large number of sequences have been recorded in the database, the human data-set was restricted to isolates from China and USA, while the avian set was restricted to isolates from duck species (wild and domesticated) from China and USA. For the sake of consistency, the swine influenza data-sets also used isolates from China and USA. The hepatitis C virus, measles and dengue viruses data-sets were obtained from Biovirus.org [38] http://biovirus.org. As with the influenza data-set, the sets of coding sequences can be obtained from the web site. The HIV data-sets were obtained from the Los Alamos HIV databases http://www.hiv.lanl.gov. In this case the sets of genomes for each HIV1 subtype (b, c and d) had to first be downloaded. Then the Gene Cutter tool on the LANL site was used to produce a set of aligned sequences which then had to be post-processed to remove the gap character ‘−’. The rotavirus A data-set was obtained from NCBI Viral Genomes as sets of segments which where first collected as complete genomes. Complete polyomavirus genomes were also obtained from NCBI Viral Genomes. The data-sets were first split into subtypes (if appropriate) and then into the different genes.

### Sequence Processing

For each of the gene data-sets, after automatically removing a small number of faulty sequences (either clearly too long or too short, or with predicted coding sequences whose lengths are not a multiple of 3) the number of sequences in each set was recorded (shown in Table 1 as *Init N*). Because duplicate sequences lengthen processing times but add no additional information and can produce artefacts in phylogenetic trees, duplicate sequences were deleted to produce the final set of sequences for each gene. (The final counts are listed in Table 1 under *Final N*). The existence of paralogues is not a problem for the small viruses analysed here. For each gene across all the species and subtypes, e.g. matrix M1 protein sequences from avian influenza A virus, a codon-based multiple sequence alignment was created across using a Python application which calls Muscle [5]. The codon-based multiple sequence alignment then became input for both the pairwise 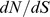 calculations and, in protein form, the MeaPED analyses.

### Computing MeaPED Scores and 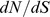


MeaPED analyses were undertaken by a computer program, ave_evol_dist.py written in the programming language Python www.python.org. MeaPED first calls Muscle to create a multiple sequence alignment, if one is not already provided, and then calls a phylogenetic tree building application to create a phylogenetic tree based on the multiple sequence alignment. The phylogenetic tree building application Phyml version 3.0 [6] was used as it is a Maximum Likelihood method but still able to process the large numbers of sequences found in some of the data-sets. Branch-length optimisation was specified. For comparison, phylogenetic-tree computations were also carried out using the Neighbour-Joining application Neighbor (from the Phylip suite [29]). Once the phylogenetic tree has been created, ave_evol_dist.py then traverses the tree to create a matrix which records the evolutionary distance between each leaf node in the tree (i.e. input sequence) and every other leaf node. Using this information, mean distances between each node/sequence and all the others can be computed, and then the mean of these means across all the (unique) sequences in the data-set for that protein. Finally, the adjusted mean of means (AMM) and adjusted mean of means per 100 aa (AMM100) were computed, as described above.

The 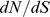 computations were undertaking using the codeml application from the PAML suite [10], with input from the codon-based multiple sequence alignments and the corresponding Phyml trees. A single 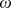 value was returned for each pairwise computation spanning both input sequences. The mean pairwise 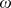 value was then computed across all the pairwise comparisons.

To estimate the evolutionary pressure on the human proteins ANT3 (gene name SLC25A6) and VDAC1, records of single nucleotide polymorphisms (SNPs) were obtained from the International HapMap Project www.hapmap.org [39]. The HapMap project has undertaken a comprehensive SNP survey across a limited number of individuals (270 in Phase II) from diverse geographic locations. HapMap’s BioMart tool returned no SNPs in the the exons of the genes encoding these two proteins. Because the HapMap methodology has involved a small number of genomes, an alternative approach was to examine the Ensembl records for the two genes www.ensembl.org [40]. In this case, use of Ensembl’s Population Comparison tool across the two protein coding transcripts for the gene SLC25A6 revealed a single non-synonymous mutation. However, use of the Population Use of the Comparison tool across the five protein coding transcripts for the VDAC1 did not yield a single non synonymous mutation.

### Statistical Methods

The Python scipy mathematical/statistical functions suite was used for the statistical computations. The linear regression function linregress was used for the omparisons of MeaPED scores (and 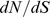) with median input sequence. Statistical comparison of the two MeaPED analyses – one using Phyml as the phylogenetic tree building application and the other using Neighbor – was carried out using the Spearman Rank Correlation function function (spearmanr). In the comparison of MeaPED versus 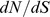 consistency (Table 4), an all against all set of comparisons of gene rankings was done for all subtypes of dengue virus, HIV, hepatitis C virus and the avian, human and swine host influenza virus. To avoid double counting, a maximum spanning tree was computed from the pairwise comparisons, such that each virus subtype appears once and there are no cycles. From the reduced set of pairwise values taken from the maximum spanning tree mean correlations of determination 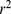 were computed, together with combined p-values based on both Stouffer’s and Fisher’s methods (see discussion in Mosteller and Bush (1954) [41]). A final note on estimating p-values from Spearman Rank Correlations. For 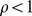, the estimated p-value returned by the spearmanr scipy function was used. However, when 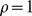 – a perfect match – the p-value is 0, even when a small number of items are being compared. This clearly overstates the significance of the match, and prevents both Stouffer and Fisher combined p-values being computed. Instead, the p-value for a perfect match involving lists of length 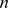 was estimated by computing the Spearman Rank Correlation of two sorted lists of unique integers of length 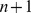, where the lists were identical except that in one list two adjacent integers had the same value (in which case the rank difference is averaged).

## Supporting Information

Table S1
**Protein Evolutionary Distances Based on Phyml - Initial and Final counts of sequences, Mean PED, Adjusted Mean PED and Adjusted Mean PED per 100 aa, and mean 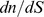.**
(PDF)

## References

[1] Drake JW (1991) A constant rate of spontaneous mutation in dna-based microbes. Proc Natl Acad Sci USA 88: 7160–7164. 1831267 10.1073/pnas.88.16.7160 PMC52253

[2] Domingo E, Holland JJ (1997) RNA virus mutations and fitness for survival. Annu Rev Microbiol 51: 151–178. 9343347 10.1146/annurev.micro.51.1.151

[3] Faith DP (1992) Conservation evaluation and phylogenetic diversity. Biol Conserv 61: 1–10.

[4] D’Erchia AM, Gissi C, Pesole G, Saccone C, Arnason U (1996) The guinea-pig is not a rodent. Nature 381: 597–600. 8637593 10.1038/381597a0

[5] Edgar RC (2004) Muscle: A multiple sequence alignment method with reduced time and space complexity. BMC Bioinformatics 5. 10.1186/1471-2105-5-113 PMC517706 15318951

[6] Guindon S, Gascuel O (2003) A simple, fast, and accurate algorithm to estimate large phylogenies by maximum likelihood. Syst Biol 52: 696–704. 14530136 10.1080/10635150390235520

[7] Saitou N, Nei M (1987) The neighbor-joining method: A new method for reconstructing phylogene- tic trees. Mol Biol Evol 4: 406–425. 3447015 10.1093/oxfordjournals.molbev.a040454

[8] Farris JS (1972) Estimating phylogenetic trees from distance matrices. Am Nat 106: 645–668.

[9] Pàl C, Papp B, Lercher MJ (2006) An integrated view of protein evolution. Nat Rev Genet 7: 337–348. 16619049 10.1038/nrg1838

[10] Yang Z (2006) Computational Molecular Evolution. Oxford University Press.

[11] Kryazhimskiy S, Plotkin JB (2008) The population genetics of dn/ds. PLoS Genet 4: e1000304. 19081788 10.1371/journal.pgen.1000304 PMC2596312

[12] Suhre K (2005) Gene and genome duplication in Acanthamoeba polyphaga Mimivirus. J Virol 79: 14095–14101. 16254344 10.1128/JVI.79.22.14095-14101.2005 PMC1280231

[13] Lemoine F, Lespinet O, Labedan B (2007) Assessing the evolutionary rate of positional orthologous genes in prokaryotes using synteny data. BMC Evol Biol 7: 237. 18047665 10.1186/1471-2148-7-237 PMC2238764

[14] Chen W, Calvo PA, Malide D, Gibbs J, Schubert U, et al (2001) A novel influenza A virus mitochondrial protein that induces cell death. Nature Med 7: 1306–1312. 11726970 10.1038/nm1201-1306

[15] Zamarin D, Garcia-Sastre A, Xiao X, Wang R, Palese P (2005) Influenza virus PB1-F2 protein induces cell death through mitochondrial ANT3 and VDAC1. PLoS Pathog 1: e4. 16201016 10.1371/journal.ppat.0010004 PMC1238739

[16] Mazur I, Anhlan D, Mitzner D, Wixler L, Schubert U, et al (2008) The proapoptotic influenza A virus protein PB1-F2 regulates viral polymerase activity by interaction with the PB1 protein. Cell Microbiol 10: 1140–1152. 18182088 10.1111/j.1462-5822.2008.01116.x

[17] Chevalier C, Bazzal AA, Vidic J, Fevrier V, Bourdieu C, et al (2010) PB1-F2 influenza A virus protein adopts a beta-sheet conformation and forms amyloid fibers in membrane environments. J Biol Chem 285: 13233–13243. 20172856 10.1074/jbc.M109.067710 PMC2857135

[18] Trifonov V, Racaniello V, Rabadan R (August 28 2009) The contribution of the PB1-F2 protein to the fitness of influenza A viruses and its recent evolution in the 2009 influenza A (H1N1) pandemic virus. PLoS Curr 1. 10.1371/currents.RRN1006 PMC2762337 20029605

[19] Donelan NR, Basler CF, Garca-Sastre A (2003) A recombinant influenza A virus expressing an RNA-binding-defective NS1 protein induces high levels of beta interferon and is attenuated in mice. J Virol 77: 13257–13266. 14645582 10.1128/JVI.77.24.13257-13266.2003 PMC296096

[20] Montserret R, Saint N, Vanbelle C, Salvay AG, Simorre JP, et al (2010) NMR structure and ion channel activity of the p7 protein from hepatitis C virus. J Biol Chem 41: 31446–31461. 10.1074/jbc.M110.122895 PMC2951219 20667830

[21] Vieyres G, Thomas X, Descamps V, Duverlie G, Patel AH, et al (2010) Characterization of the envelope glycoproteins associated with infectious hepatitis C virus. J Virol 84: 10159–10168. 20668082 10.1128/JVI.01180-10 PMC2937754

[22] Sominskaya I, Alekseeva E, Skrastina D, Mokhonov V, Starodubova E, et al (2006) Signal sequences modulate the immunogenic performance of human hepatitis C virus E2 gene. Mol Immunol 43: 1941–1952. 16442623 10.1016/j.molimm.2005.11.018

[23] Dumoulin FL, von dem Bussche A, Li J, Khamzina L, Wands JR, et al (2003) Hepatitis C virus NS2 protein inhibits gene expression from different cellular and viral promoters in hepatic and nonhepatic cell lines. Virology 305: 260–266. 12573571 10.1006/viro.2002.1701

[24] Erdtmann L, Franck N, Lerat H, Seyec JL, Gilot D, et al (2003) The hepatitis C virus NS2 protein is an inhibitor of CIDE-B-induced apoptosis. J Biol Chem 278: 18256–18264. 12595532 10.1074/jbc.M209732200

[25] Penin F (2003) Structural biology of hepatitis C virus. Clin Liver Dis 7: 1–21. 12691456 10.1016/s1089-3261(02)00066-1

[26] Chesters PM, Heritage J, McCance DJ (1983) Persistence of DNA sequences of BK virus and JC virus in normal human tissues and in diseased tissues. J Infect Dis 147: 676–684. 6302172 10.1093/infdis/147.4.676

[27] Villareal LP (1999) DNA virus contribution to host evolution. In: Esteban Domingo RGW, Holland JF, editors, Origin and Evolution of Viruses, Academic Press. pp.391–420.

[28] Le SQ, Gascuel O (2008) An improved general amino acid replacement matrix. Mol Biol Evol 25: 1307–1320. 18367465 10.1093/molbev/msn067

[29] Felsenstein J (2005) PHYLIP (Phylogeny Inference Package) version 3.6. Distributed by the au- thor, Department of Genome Sciences, University of Washington, Seattle. http://evolution. genetics.washington.edu/phylip.html.

[30] Penn O, Stern A, Rubinstein ND, Dutheil J, Bacharach E, et al (2008) Evolutionary modeling of rate shifts reveals specificity determinants in hiv-1 subtypes. PLoS Comput Biol 4: e1000214. 18989394 10.1371/journal.pcbi.1000214 PMC2566816

[31] Pfaller CK, Conzelmann KK (2008) Measles virus V protein is a decoy substrate for i*κ*b kinase α and prevents toll-like receptor 7/9-mediated interferon induction. J Virol 82: 12365–12373. 18922877 10.1128/JVI.01321-08 PMC2593327

[32] Hashiguchi T, Kajikawa M, Maita N, Takeda M, Kuroki K, et al (2007) Crystal structure of measles virus hemagglutinin provides insight into effective vaccines. Proc Natl Acad Sci USA 104: 19535–19540. 18003910 10.1073/pnas.0707830104 PMC2148324

[33] Yanagi Y, Takeda M, Ohno S (2006) Measles virus: Cellular receptors, tropism and pathogenesis. J Gen Virol 87: 2767–2779. 16963735 10.1099/vir.0.82221-0

[34] Webster RG, Bean WJ, Gorman OT, Chambers TM, Kawaoka Y (1992) Evolution and ecology of influenza A viruses. Microbiol Rev 56: 152–179. 1579108 10.1128/mr.56.1.152-179.1992 PMC372859

[35] Watt P (2006) Screening for peptide drugs from the natural repertoire of biodiverse protein folds. Nature Biotechnol 24: 177–183. 16465163 10.1038/nbt1190

[36] Dessimoz C, Gil M (2010) Phylogenetic assessment of alignments reveals neglected tree signal in gaps. Genome Biol 11: R37. 20370897 10.1186/gb-2010-11-4-r37 PMC2884540

[37] Bao Y, Bolotov P, Dernovoy D, Kiryutin B, Zaslavsky L, et al (2008) The influenza virus resource at the national center for biotechnology information. J Virol 82: 596–601. 17942553 10.1128/JVI.02005-07 PMC2224563

[38] Greene JM, Collins F, Lefkowitz EJ, Roos D, Scheuermann RH, et al (2007) National institute of allergy and infectious diseases bioinformatics resource centers: New assets for pathogen informatics. Infect Immun 75: 3212–3219. 17420237 10.1128/IAI.00105-07 PMC1932942

[39] The-International-HapMap-Consortium (2007) A second generation human haplotype map of over 3.1 million snps. Nature 449: 851–861. 17943122 10.1038/nature06258 PMC2689609

[40] Hubbard TJP, Aken BL, Beal K, Ballester B, Caccamo M, et al (2007) Ensembl 2007. Nucleic Acids Res 35: D610–D617. 17148474 10.1093/nar/gkl996 PMC1761443

[41] Mosteller F, Bush RR (1954) Selected quantitative techniques. In: Lindzey G, editor, Handbook of Social Psychology, Addison-Wesley. pp.289–334.

