# Supplementary material for: Mean Protein Evolutionary Distance: A Method for Comparative Protein Evolution and Its Application
Source: PLoS One. 2013 Apr 15;8(4):e61276. doi: 10.1371/journal.pone.0061276 (PMC3626687; doi:10.1371/journal.pone.0061276)
Supplement: Table S1 — Protein Evolutionary Distances Based on Phyml - Initial and Final counts of sequences, Mean PED, Adjusted Mean PED and Adjusted Mean PED per 100 aa, and mean . (PDF) [file pone.0061276.s001.pdf]

Supporting Table 1

For each virus type/subtype, against the proteins listed in Column 1 the mean Protein Evolutionary Distance, Adjusted Mean Protein Evolutionary Distance and Adjusted Mean Protein Evolutionary Distance per 10aa are reported in columns 5-7. Column 2 gives the median sequence length for the protein sequence, while Columns 3 and 4 indicate, respectively, the starting count of sequences and the actual number of sequences once identical sequences have been removed. The final column is the mean dN/dS value for the gene.

Table 1. Protein Evolutionary Distances Based on Phyml - Initial and Final counts of sequences, Mean PED, Adjusted Mean PED and Adjusted Mean PED per 100aa, and mean dn/dS

**Dengue virus**							
Dengue virus type1							
Protein	Med.Len	Init N	Final N	Mean	Adj. Mean	Adj. Mean per 100aa	dnds
NS2a	654.0	651	323	0.0394	0.0196	0.0090	0.0925
M	221.3	651	186	0.0219	0.0063	0.0086	0.0622
NS4a	381.0	649	246	0.0254	0.0096	0.0076	0.0592
C	300.0	651	149	0.0297	0.0068	0.0068	0.1883
NS2b	390.0	651	258	0.0219	0.0087	0.0067	0.0415
2K	69.0	622	60	0.0158	0.0015	0.0066	0.0336
NS1	1056.0	651	392	0.0288	0.0174	0.0049	0.0560
NS4b	747.0	651	304	0.0158	0.0074	0.0030	0.0383
E	1489.4	651	435	0.0195	0.0130	0.0026	0.0525
NS5	2697.0	633	472	0.0212	0.0158	0.0018	0.0512
NS3	1857.0	651	465	0.0131	0.0093	0.0015	0.0281
Dengue virus type2							
Protein	Med.Len	Init N	Final N	Mean	Adj. Mean	Adj. Mean per 100aa	dnds
M	225.0	615	180	0.0343	0.0100	0.0134	0.0826
NS2a	654.0	615	357	0.0480	0.0278	0.0128	0.0675
NS4a	381.0	580	268	0.0327	0.0151	0.0119	0.0507
2K	69.0	615	59	0.0185	0.0018	0.0077	0.0121
NS2b	390.0	615	256	0.0237	0.0099	0.0076	0.0443
C	300.0	615	188	0.0222	0.0068	0.0068	0.0690
NS4b	744.0	613	358	0.0267	0.0156	0.0063	0.0371
NS1	1056.0	614	403	0.0319	0.0209	0.0059	0.0539
E	1485.0	615	447	0.0249	0.0181	0.0037	0.0428
NS5	2700.0	615	515	0.0351	0.0294	0.0033	0.0503
NS3	1854.0	615	467	0.0215	0.0164	0.0027	0.0296

Dengue virus type3							
Protein	Med.Len	Init N	Final N	Mean	Adj. Mean	Adj. Mean per 100aa	dnds
2K	69.0	356	45	0.0315	0.0040	0.0173	0.0457
NS4a	381.0	355	161	0.0284	0.0129	0.0101	0.0777
NS2a	654.0	356	213	0.0250	0.0150	0.0069	0.1079
M	225.0	356	112	0.0151	0.0048	0.0063	0.0584
C	300.0	356	111	0.0190	0.0059	0.0059	0.0986
NS1	1056.0	356	235	0.0179	0.0118	0.0034	0.0728
E	1479.0	356	270	0.0216	0.0164	0.0033	0.0448
NS2b	390.0	356	150	0.0059	0.0025	0.0019	0.0399
NS5	2700.0	356	300	0.0163	0.0138	0.0015	0.0509
NS4b	744.0	356	211	0.0062	0.0037	0.0015	0.0184
NS3	1857.0	356	278	0.0098	0.0077	0.0012	0.0270

Dengue virus type4							
Protein	Med.Len	Init N	Final N	Mean	Adj. Mean	Adj. Mean per 100aa	dnds
2K	69.0	44	15	0.0671	0.0229	0.0995	0.0854
NS2a	654.0	44	31	0.0462	0.0325	0.0149	0.0980
NS4a	381.0	44	28	0.0190	0.0121	0.0095	0.0389
NS2b	390.0	44	30	0.0137	0.0093	0.0072	0.0458
M	225.0	44	24	0.0097	0.0053	0.0070	0.0197
C	297.0	43	23	0.0125	0.0067	0.0068	0.0525
NS1	1056.0	44	34	0.0296	0.0229	0.0065	0.0842
E	1485.0	44	35	0.0180	0.0143	0.0029	0.0454
NS4b	735.0	44	32	0.0095	0.0069	0.0028	0.0269
NS3	1854.0	44	35	0.0103	0.0082	0.0013	0.0267
NS5	2700.0	44	38	0.0135	0.0117	0.0013	0.0447

**Human Immunodeficiency Virus type 1**							
HIV1 subtype b							
Protein	Med.Len	Init N	Final N	Mean	Adj. Mean	Adj. Mean per 100aa	dnds
VPU	247.9	1018	700	0.5390	0.3706	0.4575	0.2750
VPR	290.9	1001	692	0.5365	0.3709	0.3863	0.3525
NEF	626.6	795	624	0.9122	0.7160	0.3459	0.3616
TAT	306.1	1021	722	0.4233	0.2993	0.2963	0.9970
REV	351.0	1023	724	0.3848	0.2723	0.2348	0.8367
VIF	579.2	1016	754	0.3996	0.2966	0.1545	0.4192
ENV	2575.5	975	875	0.7628	0.6845	0.0799	0.4389
GAG	1509.0	996	810	0.3543	0.2881	0.0575	0.1367
POL	3015.8	984	896	0.2816	0.2564	0.0256	0.1336

HIV1 subtype c							
Protein	Med.Len	Init N	Final N	Mean	Adj. Mean	Adj. Mean per 100aa	dnds
VPU	259.0	478	434	0.7277	0.6607	0.7683	0.4349
TAT	306.1	492	455	0.5266	0.4870	0.4822	0.6731
NEF	628.6	462	430	0.6546	0.6092	0.2943	0.4080
VPR	290.9	491	456	0.2610	0.2424	0.2525	0.2520
REV	369.0	488	453	0.2690	0.2497	0.2014	0.7694
VIF	579.0	488	457	0.2679	0.2509	0.1307	0.3377
ENV	2571.3	441	424	0.9083	0.8733	0.1021	0.4265
GAG	1486.3	457	425	0.4074	0.3789	0.0770	0.2321
POL	3007.4	436	415	0.2909	0.2769	0.0277	0.1397

HIV1 subtype d							
Protein	Med.Len	Init N	Final N	Mean	Adj. Mean	Adj. Mean per 100aa	dnds
TAT	305.9	59	52	0.5020	0.4425	0.4338	1.1104
VPU	246.6	60	53	0.3849	0.3400	0.4198	0.4076
VPR	290.9	60	53	0.2609	0.2304	0.2401	0.3767
REV	351.0	58	51	0.2127	0.1870	0.1613	0.9350
NEF	626.1	52	45	0.3560	0.3080	0.1488	0.3960
VIF	579.1	60	53	0.2072	0.1830	0.0953	0.3859
ENV	2555.1	59	52	0.6303	0.5555	0.0654	0.4816
GAG	1499.7	56	50	0.2102	0.1877	0.0377	0.2545
POL	3010.5	57	53	0.1441	0.1340	0.0134	0.1889

**Hepatitis C Virus**							
Hepatitis C virus type 1							
Protein	Med.Len	Init N	Final N	Mean	Adj. Mean	Adj. Mean per 100aa	dnds
p7	189.0	804	749	0.3524	0.3283	0.5211	0.1158
NS2	651.0	804	778	0.5698	0.5514	0.2541	0.0974
E1	576.1	804	764	0.4907	0.4663	0.2428	0.0878
NS4a	162.0	804	733	0.1388	0.1265	0.2343	0.0698
E2	1089.3	804	780	0.7518	0.7294	0.2009	0.0761
F	485.0	630	581	0.3161	0.2915	0.1810	4.6603
NS4b	783.0	804	774	0.3403	0.3276	0.1255	0.0397
NS5a	1342.8	804	779	0.4860	0.4709	0.1051	0.0859
NS5b	1769.7	743	733	0.2824	0.2787	0.0471	0.0866
NS3	1893.0	804	782	0.3045	0.2962	0.0469	0.0349
C	573.0	804	754	0.0481	0.0451	0.0236	0.0482

Hepatitis C virus type 2							
Protein	Med.Len	Init N	Final N	Mean	Adj. Mean	Adj. Mean per 100aa	dnds
p7	189.0	49	46	0.2878	0.2702	0.4289	0.1038
NS4a	162.0	49	46	0.2110	0.1980	0.3668	0.0741
F	435.2	47	44	0.4614	0.4319	0.2742	3.8434
E1	576.1	49	49	0.3622	0.3622	0.1886	0.0954
E2	1100.9	49	49	0.5309	0.5309	0.1447	0.0669
NS2	651.0	49	49	0.2969	0.2969	0.1368	0.1157
NS5a	1396.2	49	49	0.3963	0.3963	0.0851	0.0920
NS4b	783.0	49	49	0.0952	0.0952	0.0365	0.0303
C	573.0	49	48	0.0619	0.0606	0.0317	0.0975
NS5b	1772.2	46	46	0.1481	0.1481	0.0251	0.0773
NS3	1893.0	49	49	0.1259	0.1259	0.0200	0.0300
Hepatitis C virus type 3							
Protein	Med.Len	Init N	Final N	Mean	Adj. Mean	Adj. Mean per 100aa	dnds
p7	189.0	10	10	0.1722	0.1722	0.2734	0.1390
F	437.5	6	6	0.1369	0.1369	0.0957	3.0775
NS4a	162.0	10	9	0.0565	0.0508	0.0941	0.0354
E1	576.3	10	10	0.1796	0.1796	0.0935	0.1241
E2	1107.0	10	10	0.2694	0.2694	0.0730	0.1977
NS2	651.0	10	10	0.1367	0.1367	0.0630	0.1058
NS5a	1355.7	10	10	0.1993	0.1993	0.0441	0.1338
C	573.0	10	10	0.0673	0.0673	0.0352	0.1054
NS4b	783.0	10	10	0.0853	0.0853	0.0327	0.0904
NS5b	1773.0	7	7	0.1091	0.1091	0.0185	0.1143
NS3	1893.0	10	10	0.0683	0.0683	0.0108	0.0599
Hepatitis C virus type 4							
Protein	Med.Len	Init N	Final N	Mean	Adj. Mean	Adj. Mean per 100aa	dnds
p7	189.0	30	30	0.3085	0.3085	0.4897	0.0637
NS4a	162.0	30	30	0.1081	0.1081	0.2002	0.0428
NS2	651.0	30	30	0.3968	0.3968	0.1829	0.0802
F	547.2	29	29	0.2861	0.2861	0.1555	5.3335
E1	576.0	30	30	0.2309	0.2309	0.1203	0.0499
E2	1089.8	30	30	0.3681	0.3681	0.1014	0.0801
NS5a	1335.6	30	30	0.2518	0.2518	0.0566	0.0725
NS4b	783.0	30	30	0.0720	0.0720	0.0276	0.0307
NS5b	1773.1	21	21	0.1471	0.1471	0.0249	0.0914
C	573.0	30	30	0.0233	0.0233	0.0122	0.0400
NS3	1893.0	30	30	0.0747	0.0747	0.0118	0.0422

Hepatitis C virus type 6							
Protein	Med.Len	Init N	Final N	Mean	Adj. Mean	Adj. Mean per 100aa	dnds
p7	189.0	60	56	0.8345	0.7788	1.2362	0.0559
F	409.6	47	44	0.7051	0.6601	0.5281	3.6124
NS4a	162.0	60	56	0.2436	0.2274	0.4211	0.0254
E1	576.0	60	57	0.5861	0.5568	0.2900	0.0338
NS2	651.0	60	57	0.4912	0.4666	0.2150	0.0401
E2	1097.1	60	57	0.6425	0.6104	0.1672	0.0311
NS5a	1354.6	60	57	0.5654	0.5371	0.1191	0.0404
NS4b	783.0	60	57	0.2674	0.2540	0.0973	0.0299
C	573.0	60	57	0.1171	0.1112	0.0582	0.0562
NS5b	1773.0	60	60	0.3067	0.3067	0.0519	0.0767
NS3	1893.0	60	57	0.1646	0.1564	0.0248	0.0155

**Influenza**							
Avian Influenza							
Protein	Med.Len	Init N	Final N	Mean	Adj. Mean	Adj. Mean per 100aa	dnds
NA	1396.7	1465	1178	1.9651	1.5801	0.3369	0.0543
HA	1691.5	1442	1241	1.6950	1.4588	0.2582	0.0361
PB1-F2	265.2	1074	522	0.2287	0.1112	0.1235	1.8693
NS1	683.7	1475	1009	0.2681	0.1834	0.0797	0.1157
NS2	363.0	1454	739	0.1155	0.0587	0.0485	0.1137
M2	291.0	1427	568	0.0732	0.0291	0.0300	0.4159
M1	756.0	1470	996	0.0197	0.0134	0.0053	0.0251
PA	2148.0	1454	1241	0.0350	0.0299	0.0042	0.0256
NP	1494.0	1455	1152	0.0239	0.0189	0.0038	0.0183
PB1	2271.1	1448	1212	0.0212	0.0178	0.0024	0.0167
PB2	2277.0	1443	1244	0.0207	0.0178	0.0024	0.0150
Swine Influenza							
Protein	Med.Len	Init N	Final N	Mean	Adj. Mean	Adj. Mean per 100aa	dnds
PB1-F2	201.8	131	59	1.3888	0.6255	1.2029	2.9160
NA	1405.9	221	185	1.0791	0.9033	0.1926	0.0833
HA	1696.4	223	184	1.0351	0.8540	0.1509	0.0802
M2	291.0	209	103	0.1331	0.0656	0.0676	0.6504
NS1	663.0	220	156	0.1687	0.1196	0.0546	0.2701
NS2	363.0	210	119	0.0918	0.0520	0.0430	0.2072
NP	1494.0	214	168	0.0614	0.0482	0.0097	0.0491
PA	2148.0	223	186	0.0810	0.0675	0.0094	0.0485
M1	756.0	222	152	0.0275	0.0188	0.0075	0.0444
PB2	2277.0	223	186	0.0675	0.0563	0.0074	0.0430

Protein	Med.Len	Init N	Final N	Mean	Adj. Mean	Adj. Mean per 100aa	dnds
PB1	2271.1	221	182	0.0578	0.0476	0.0063	0.0314

Human Influenza							
Protein	Med.Len	Init N	Final N	Mean	Adj. Mean	Adj. Mean per 100aa	dnds
HA	1697.3	3368	2159	1.5015	0.9625	0.1701	0.1042
NA	1407.3	3357	1777	1.1722	0.6205	0.1323	0.1258
PB1-F2	237.8	1932	344	0.6240	0.1111	0.1235	1.3072
NS1	680.7	3364	1140	0.2652	0.0899	0.0391	0.2740
M2	290.8	3359	539	0.1301	0.0209	0.0215	0.4055
NS2	363.0	3349	624	0.0995	0.0185	0.0153	0.1124
NP	1494.0	3345	1656	0.1308	0.0648	0.0130	0.0827
PA	2148.0	3339	2090	0.0691	0.0432	0.0060	0.0640
M1	756.0	3363	913	0.0542	0.0147	0.0058	0.0756
PB2	2277.0	3338	2190	0.0624	0.0409	0.0054	0.0590
PB1	2271.1	3343	2143	0.0448	0.0287	0.0038	0.0496

**Measles**							
Protein	Med.Len	Init N	Final N	Mean	Adj. Mean	Adj. Mean per 100aa	dnds
V	900.4	23	14	0.0407	0.0248	0.0083	1.0998
C	561.0	30	16	0.0276	0.0147	0.0079	0.2756
P	1524.0	34	23	0.0387	0.0262	0.0052	0.6530
N	1578.0	34	22	0.0236	0.0153	0.0029	0.2288
M	1008.0	33	22	0.0141	0.0094	0.0028	0.3392
H	1854.5	34	23	0.0246	0.0166	0.0027	0.7055
F	1657.8	34	22	0.0126	0.0081	0.0015	0.4244
L	6552.0	34	29	0.0119	0.0101	0.0005	0.1804

**Polyomavirus**							
Protein	Med.Len	Init N	Final N	Mean	Adj. Mean	Adj. Mean per 100aa	dnds
AGNO	199.3	528	38	0.0394	0.0028	0.0043	0.4549
VP1	1089.0	530	124	0.0425	0.0099	0.0027	0.1217
VP3	699.0	528	52	0.0334	0.0033	0.0014	0.1531
VP2	1056.0	528	57	0.0238	0.0026	0.0007	0.1554
ST	519.0	530	48	0.0109	0.0010	0.0006	0.1269
LT	2087.6	526	138	0.0089	0.0023	0.0003	0.0328

Rotavirus							
Protein	Med.Len	Init N	Final N	Mean	Adj. Mean	Adj. Mean per 100aa	dnds
VP7	977.2	135	98	0.2149	0.1560	0.0478	0.0609
NSP1	1458.9	129	94	0.2253	0.1641	0.0338	0.1122
NSP4	525.0	134	84	0.0534	0.0335	0.0191	0.1037
NSP5	591.3	131	67	0.0534	0.0273	0.0139	0.1339
NSP3	931.7	118	78	0.0466	0.0308	0.0099	0.0613
NSP2	951.0	122	71	0.0488	0.0284	0.0090	0.0461
VP4	2325.2	135	102	0.0907	0.0685	0.0088	0.0401
VP3	2505.0	130	98	0.0624	0.0471	0.0056	0.0537
VP6	1189.7	130	82	0.0172	0.0109	0.0027	0.0131
VP2	2675.1	131	98	0.0307	0.0230	0.0026	0.0214
VP1	3260.5	135	113	0.0246	0.0206	0.0019	0.0345
